# The synergistic ameliorative activity of peroxisome proliferator‐activated receptor‐alpha and gamma agonists, fenofibrate and pioglitazone, on hippocampal neurodegeneration in a rat model of insulin resistance

**DOI:** 10.1002/ibra.12059

**Published:** 2022-08-08

**Authors:** Olumayowa K. Idowu, Olushola O. Oluyomi, Oluwatomisin O. Faniyan, Olufunke O. Dosumu, Oluwole B. Akinola

**Affiliations:** ^1^ Department of Anatomy University of Ilorin Ilorin Nigeria; ^2^ Department of Anatomy Osun State University Osogbo Nigeria; ^3^ Department of Physiology, School of Bioscience and Veterinary Medicine University of Camerino Camerino Italy; ^4^ Department of Anatomy University of Lagos Lagos Nigeria

**Keywords:** cognitive impairment, high‐fat diet/streptozotocin, PPARα/γ agonists

## Abstract

Insulin resistance (IR) is a risk factor for metabolic disorders and neurodegeneration. Peroxisome proliferator‐activated receptor (PPAR) agonists have been proven to mitigate the neuronal pathology associated with IR. However, the synergetic efficacy of these agonists is yet to be fully described. Hence, we aimed to investigate the efficacy of PPARα/γ agonists (fenofibrate and pioglitazone) on a high‐fat diet (HFD) and streptozotocin (STZ)‐induced hippocampal neurodegeneration. Male Wistar rats (200 ± 25 mg/body weight [BW]) were divided into five groups. The experimental groups were fed on an HFD for 12 weeks coupled with 5 days of an STZ injection (30 mg/kg/BW, i.p) to induce IR. Fenofibrate (FEN; 100 mg/kg/BW, orally), pioglitazone (PIO; 20 mg/kg/BW, orally), and their combination were administered for 2 weeks postinduction. Behavioral tests were conducted, and blood was collected to determine insulin sensitivity after treatment. Animals were killed for assessment of oxidative stress, cellular morphology characterization, and astrocytic evaluation. HFD/STZ‐induced IR increased malondialdehyde (MDA) levels and decreased glutathione (GSH) levels. Evidence of cellular alterations and overexpression of astrocytic protein was observed in the hippocampus. By contrast, monotherapy of FEN and PIO increased the GSH level (*p* < 0.05), decreased the MDA level (*p* < 0.05), and improved cellular morphology and astrocytic expression. Furthermore, the combined treatment led to improved therapeutic activities compared to monotherapies. In conclusion, FEN and PIO exerted a therapeutic synergistic effect on HFD/STZ‐induced IR in the hippocampus.

## Graphical abstract

Induction of symptoms of insulin insensitivity with the concomitant administration of streptozotocin (STZ) and a high‐fat diet (HFD) increased the fasting glucose level. This alters the neural activities and morphology of the hippocampus via negative activation of Peroxisome proliferator‐activated receptor (PPARs)‐regulated signaling pathways needed for synaptic plasticity and neuronal survival. However, the use of fenofibrate and pioglitazone (PPARs agonists) results in competition for similar active sites and leads to modulation of the respective signaling pathways altered by insulin insensitivity to mitigate alterations of the hippocampus and improve its functionality and neuronal survival.

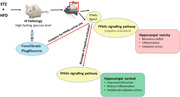

## 1 INTRODUCTION

Neurodegeneration is a spectrum of pathological conditions resulting from the progressive loss of the neural structure.^1^ This causes a gradual decline in neural functioning, including motor, behavioral, and higher cognitive functions.^1^, ^2^, ^3^ Evidence of neurodegeneration has been reported as one of the leading causes of many neurological disorders.^1^, ^2^, ^4^ Pathological conditions involving mitochondria dysfunction, esterase of synaptic neurotransmitters, alterations of nuclei materials, and deposition of toxic protein in the microfilament of the neurons have been strongly associated with the onset and progressive loss of neuronal structures and neural plasticity responsible for the cognitive function of the brain, especially in the hippocampus.^4^, ^5^, ^6^ Despite this vast knowledge, the main cause of neurodegeneration is yet to be fully described.

Adequate release of ATP via glucose metabolism by the neuronal mitochondria has been reported extensively to enhance positive neuroplasticity, learning, and reinforcement of the neural circuit,^7^, ^8^, ^9^ particularly in the hippocampus and other cortical areas of the brain, involved with cognition.^10^ Recently, incidences of insulin resistance (IR), a pathological condition associated with diabetes that triggers the insensitivity of cells to insulin, have been receiving considerable attention as a pointer to the onset and severity of neurodegeneration‐induced dementia experienced in diabetes mellites (DM) patients.^11^, ^12^, ^13^ The decline in neuronal ATP, possibly due to mitochondrial dysfunction and insensitivity of neurons to insulin, has been pinpointed as one of the mechanisms contributing to the onset of neurodegeneration and associated pathological conditions including dementia.^9^, ^14^, ^15^, ^16^ As a result, considerable attention is being focused on developing preclinical models that best describe  these symptoms, so as to fully characterize the mechanism underlying the onset and progression of the pathological conditions.

Over the years, preclinical research has been focused on the use of a singular technique to mimic the pathologies associated with IR‐triggered type 2 DM.^17^, ^18^, ^19^ A high‐fat diet (HFD), a specially compounded fat‐rich chow that induces impaired glucose metabolism by simultaneously decreasing the mRNA expression of pancreatic lipase and colipase and increasing the expression of brown adipose tissue uncoupling protein 1, has been widely reported and used to trigger pathology that mimics IR in rodents.^20^, ^21^, ^22^ For instance, long‐time exposure to HFD was linked to the induction of dyslipidemia and a spectral of pathological conditions including obesity, pancreatitis, IR, and glucose intolerance in mice.^23^ Also, streptozotocin (STZ), a glucosamine‐nitrosourea compound that damages the DNA and is selectively toxic to the insulin‐producing (beta islet) cells of the pancreas, has been widely documented to induce the pathology of hyperglycemia in research models. However, recent findings have suggested that the use of combined techniques could best mimic the spectra of pathology induced by IR on several signaling pathways including the peroxisome proliferator‐activated receptor (PPAR)‐regulated pathway.^24^, ^25^, ^26^

The PPARs coordinate the signaling cascade and have been hypothesized to be major signaling pathways altered by IR. PPARs are nuclear receptors that modulate gene expression involved in cell differentiation, immune function, lipid metabolism, glucose control, and energy homeostasis.^27^, ^28^, ^29^ Furthermore, brain regions associated with higher cognitive functions and the need for high energy consumption (via glucose metabolism) have been linked to the accumulation of PPARs.^30^, ^31^ Similarly, isoforms of PPARs located on glial cells have been linked to various peripheral and neuronal protective downstream activations.^32^, ^33^ PPAR gamma (PPARγ), specifically expressed in microglia and astrocytes, regulates the activities of anti‐ and pro‐inflammatory genes and the production of pro‐inflammatory cytokines during cellular injury.^33^, ^34^, ^35^ PPAR beta (PPARβ) expressed in the oligodendrocytes has been linked to axonal myelination needed for neural connectivity across different brain centers.^29^

Alterations in the hepatic mitochondrial and peroxisomal beta‐oxidation signaling cascade regulated by PPARα have been regarded as a target for high fat intake in type 2 DM patients. Moreso, in vivo studies, has reported that HFD induces upregulation of the PPARγ, causing fatty acid synthesis via the activation of lipogenic factors and disruption of glucose and lipid metabolism.^36^, ^37^ In a similar mechanism, STZ activates PPARα and PPARβ, leading to the disruption of lipoprotein synthesis, carbohydrate metabolism, and mitochondrial function, and inflammation in the liver, pancreas adipose tissue, and hepatic tissue.^38^, ^39^, ^40^ Therapeutically, agonists of these receptors such as fenofibrate (FEN) and pioglitazone (PIO) have been extensively reported to play a protective role in the liver, skin, kidney, lungs, and gastrointestinal system as monotherapy against IR pathology by increasing mitochondrial beta‐oxidation coupled with reduced lipogenesis (PPARα), suppressing the production of inflammatory cytokine (interleukin‐6 and interleukin‐8) and chemokines (CXCLs) and adiponectin expression and decreasing IR (PPARγ).^41^, ^42^, ^43^, ^44^ However, the role of fenofibrate and pioglitazone as monotherapy and/or combined therapy in IR‐induced hippocampal neurodegeneration, cognitive behavior, and astrocytic response is yet to be fully described. Therefore, this study aimed to investigate the synergistic ameliorative activity of fenofibrate and pioglitazone agonists on hippocampal structure and function in HFD‐ and STZ‐induced rat models of IR.

## 2 MATERIALS AND METHOD

### 2.1 Chemicals

Fenofibrate and pioglitazone (PIO) were procured from Bharat Parenterals Ltd., Gujarat, India, and Micro Labs Ltd., India, respectively. STZ was acquired from Sigma‐Aldrich Company.

### 2.2 Production of HFD

HFD was produced at a reliable indigenous feed‐mill in Ilorin, Nigeria. The diet was composed of 5 kg of palm kernel cake, 5.5 kg of maize, 0.5 kg of wheat offal, 5.5 kg of groundnut cake, 0.5 kg of bone meal, 0.5 kg of fish meal, 0.025 kg of methionine, 0.025 kg of lysine, 0.0625 kg of industrial salt, 0.0625 kg of broiler premix, and 12.5 kg of toasted soya meal.^45^, ^46^

### 2.3 Experimental design

Male adult Wistar rats with a body weight range of 180–220 g were obtained from the Animal facility of the University of Ilorin. The animals were housed in normal home cages, maintained under standardized conditions of 12 /12 h of a light/dark cycle, and received adequate care in the animal holding section of the Faculty of Basic Medical Sciences, University of Ilorin. They were allowed to acclimatize at a temperature of 23± 5°C for 14 days, during which they were fed with standard pelletized feed and water ad libitum, before the commencement of the study. The animals were randomized into two major groups (control and IR‐induced groups). The control group (*n* = 5) was fed with pelletized rat feed and distilled water. Animals recruited for the IR‐induced pathology were fed with HFD throughout the experiment (12 weeks).^47^ At Week 3, the animals were intraperitoneally administered 30 mg/kg body weight STZ dissolved in cold sodium citrate buffer (0.1 M, pH 7.4) for 5 consecutive days (a low dose of STZ was administered to induce hypoinsulinemia ^48^ since the administration of HFD for more than 8 weeks has been reported to induce IR^49^, ^50^). At Week 9, the animals were then screened for IR pathology by assessing the fasting blood glucose (FBG) level using an Accu‐chek glucometer. An FBG level above 126 mg/dl was considered to indicate hyperglycemia. Twenty animals showing the pathology were then randomized into four groups (*n* = 5): HFD/STZ, HFD/STZ/FEN, HFD/STZ/PIO, and HFD/STZ/FEN/PIO.

**Figure 1:**
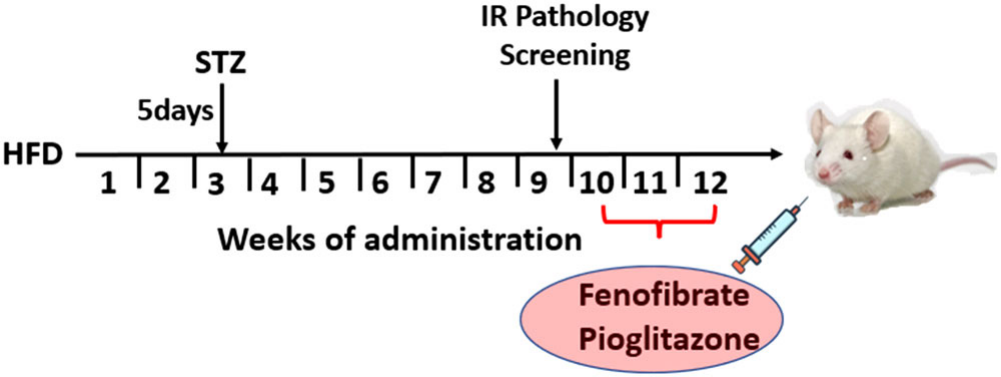
Experimental design [Color figure can be viewed at wileyonlinelibrary.com]

HFD/STZ groups were placed on an HFD for the remaining duration of the experiment. At Week 10, HFD/STZ/FEN, HFD/STZ/PIO, and HFD/STZ/FEN/PIO groups were subjected to intragastric gavage administration of FEN (100 mg/kg body weight)^51^, ^52^ and/or PIO (20 mg/kg body weight)^53^, ^54^ for 14 days consecutively^55^, ^56^ (Figure 1). After the administration, the animals were fasted overnight and anesthetized by an intraperitoneal injection of ketamine (10 mg/kg body weight).^57^ Thoracic dissection was performed to access the heart for blood collection. The hippocampal tissues were harvested, homogenized, and freeze‐preserved for biochemical analysis. For histological analysis, animals were intracardially perfused using 0.9% normal saline, followed by 4% paraformaldehyde.

### 2.4 Assay for IR

IR was assessed using the homeostatic model assessment of IR (HOMA‐IR); blood samples were collected via cardiac puncture at the right ventricle and stored in heparinized tubes and then cryocentrifuged (4°C) at 3000 rpm for 5 min. 0.5 ml of the plasma was pipetted for estimation of fasting glucose and fasting plasma insulin. The fasting plasma glucose and fasting plasma insulin levels were measured using the glucose oxidase method according to Ambade et al.^58^ and the insulin ELISA kit for rats, respectively. HOMA‐IR was calculated with the glucose concentration measured in mmol/L and insulin in μIU.^59^

$$\mathrm{HOMA}-\mathrm{IR}=\frac{\left(\text{FPI}\times \text{FPG}\right)}{22.5}.$$

### 2.5 Y‐maze test

The Y‐maze test was conducted to evaluate the spatial memory of all the experimental rats. An initial 3 days of training were conducted before the test, which was recorded for analysis. The Y‐maze apparatus is a three‐armed Y‐shaped chamber labeled A, B, and C, which are symmetrically separated at 120°. Each rat was placed at the center of the apparatus and allowed to freely explore the three arms for 8 min. The number of correct alternations (ABC, BCA, or CAB) was recorded and the percentage alternation was calculated as an index for spatial memory.^60^

$$\%\mathrm{Spontaneous}\;\mathrm{alternation}=\frac{\text{Number of right decisions}}{\text{Total number of armentries}}.$$

### 2.6 Elevated plus maze test

An elevated plus maze test was conducted to investigate the level of anxiety in the rats. The plus‐shaped maze consists of two opposing open arms (length: 40 cm, width: 9 cm) and two opposing wall‐enclosed arms (wall height: 15 cm) extending off a center square (9 cm per side) to form a plus shape. Each rat was placed at the center of the maze and given 10 min to explore the four arms of the maze. The arms entry ratio, time spent in each arm, and open arm rearing were recorded and calculated as indices for anxious behavior.^61^

### 2.7 Evaluation of oxidative stress/antioxidant levels

The hippocampi were carefully removed from the medial temporal lobe of the brain, homogenized in 30% of sucrose, and kept frozen. The homogenates were centrifuged at 14,000 rpm for 15 min at 4°C. The supernatant was collected for the assessment of MDA and GSH levels. The amount of MDA formed in each of the samples was assessed by measuring the optical density of the supernatant at 532 nm as described by Olufunke et al.^6^, ^62^ Glutathione (GSH) level was assayed by measuring the optical density of the supernatant at 412 after the addition of Ellman's reagent.^63^, ^64^

### 2.8 Histological and immunohistochemical studies

Histological and Immunohistochemical analyses were conducted to evaluate histoarchitectural changes in the hippocampus. The animals were perfused transcardially with 0.1 M PBS (pH 7.4) and 10% formalin in 0.1 M PBS (pH 7.4) as described by Olufunke et al. The hippocampal tissues were immediately fixated in 10% buffered formalin. The tissues were then dehydrated, embedded in paraffin, and sections of 5 µm thickness were obtained using a rotatory microtome. Each section was stained with hematoxylin and eosin (H&E) dyes for basic histological demonstration Fischer et al.^6^, ^65^ Sections were processed to demonstrate the Nissl substance as described by Zhou et al.^66^ and the neurofibril proteins using the Bielchowsky silver stain as described by Mirra.^67^

For astrocytic expression, the immunohistochemistry protocol was carried out according to the protocol described by Olufunke.^6^ In summary, sections were blocked in Pbs‐based 1% BSA and 0.1% Triton X solution for 1 h at room temperature and incubated in the primary antibody (Glial fibrillary acid protein [GFAP]‐AP32987SU‐N‐ dilution ratio 1:100) overnight at 4°C and then incubated in the secondary antibody for 2 h at room temperature. All samples were observed, and images were acquired at ×400 magnification using a Leica ICC50 light microscope.

### 2.9 Statistical analysis

Data collected were analyzed, and graphs were drawn using Graph Pad Prism 7 software (Graph pad software Inc.). Multiple comparisons were performed using a one‐way analysis of variance with the Tukey test. Data were reported as mean ± SEM. The significance of the analysis was set at *p* < 0.05.

## 3 RESULTS

### 3.1 PPARα/γ agonists lowered FBG in insulin‐resistant rats

**Figure 2:**
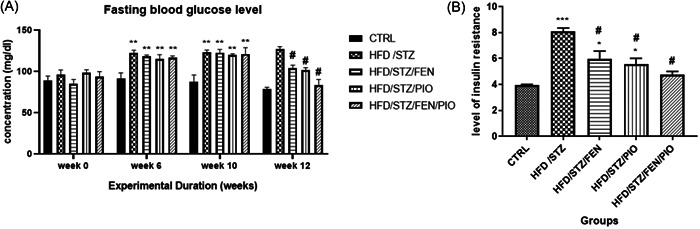
Fasting blood glucose level (A) and level of insulin resistance (B). Week 0: initial fasting blood glucose levels. Week 6: before the administration of STZ. Week 10: before the commencement of treatment, and Week 12: after 2 weeks of treatment. (*p < 0.05, **p < 0.01, ***p < 0.001 compared to the control group, ^#^ *p* < 0.05, ^##^ *p* < 0.01 compared to HFD/STZ). FEN,  fenofibrate; HFD, high‐fat diet; PIO, pioglitazone; STZ, streptozotocin.

There were no statistically significant differences in FBG levels across the groups at Week 0. From Weeks 6 to 10 of induction, there was a statistically significant increase (*p* < 0.01) in FBG levels in all induced groups compared to the control group. However, after 2 weeks of administration of PPAR agonists, there was a statistically significant decrease (*p* < 0.05) in FBG levels of all the adjuvant‐treated groups compared to the HFD/STZ group. Moreover, the HFD/STZ/FEN/PIO group showed markedly (*p* < 0.01) lower FBG levels compared to the HFD/STZ/FEN and HFD/STZ/PIO groups (Figure 2A).

### 3.2 PPARα/γ agonists enhanced insulin sensitivity in insulin‐resistant rats

The results revealed that all the adjuvant (PPARα and PPARγ agonist)‐treated groups showed a statistically (*p* < 0.05) significant decrease in IR levels compared to the HFD/STZ group. Moreover, the HFD/STZ/FEN and HFD/STZ/PIO groups showed a statistically (*p* < 0.05) significant increase in IR levels compared to the control group (Figure 2B).

### 3.3 PPARα/γ agonists reduced anxiety levels and improved spatial memory in insulin‐resistant rats

**Figure 3:**
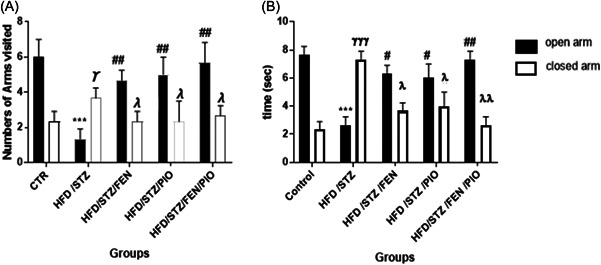
Arms entry ratio (A) and time spent in each arm (B). The HFD/STZ group made fewer entries and spent significantly less time (* *p* < 0.05) in the open arms, and more entries and time (^ϒ^ *p* < 0.05) in the closed arm when compared with the control group. HFD/STZ/FEN, HFD/STZ/PIO, and HFD/STZ/FEN/PIO groups made more entries and spent significantly more time (**^#^** *p* < 0.05, ^##^ *p* < 0.01) in the open arms, and fewer entries and time (^λ^ *p* < 0.05) in the closed arm when compared with the HFD/STZ group. FEN,  fenofibrate; HFD, high‐fat diet; PIO, pioglitazone; STZ, streptozotocin.

**Figure 4:**
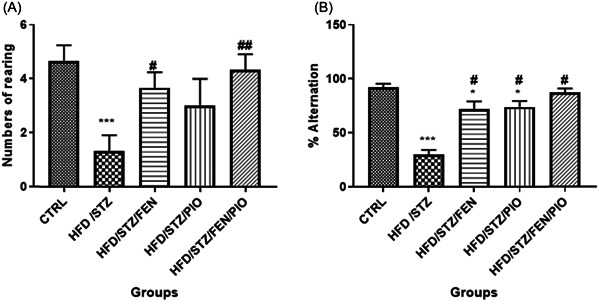
Open arm rearing (A); there is a statistically significant decrease in the numbers of open arm rearing in the HFD/STZ group compared to the control group. However, PPAR‐treated groups showed no statistically significant differences in the numbers of rearing compared to the control group, but showed a statistically significant increase in the numbers of rearing compared to the HFD/STZ‐treated group. Percentage alternation (B); there is a statistically significant percentile decrease in the Y‐maze test performance in all treated groups, except HFD/STZ/FEN/PIO, compared to the control group. Also, all adjuvant‐treated groups showed a statistically significant percentile increase in Y maze test performance compared to the HFD/STZ‐treated group (*p < 0.05, ***p < 0.001 compared to the control group, ^#^ *p* < 0.05, ^##^ *p* < 0.01 compared to the HFD/STZ group). FEN,  fenofibrate; HFD, high‐fat diet; PIO, pioglitazone; STZ, streptozotocin.

Evidence of anxiety‐like behavior in the elevated plus maze test has been linked to impaired working memory.^68^ The HFD/STZ group showed an alteration in the arm entry ratio (Figure 3A) and the total time spent in each arm (Figure 3B), as revealed by a higher preference for the closed arm (*p* < 0.05). Furthermore, there was a statistically (*p* < 0.05) significant decrease in the number of open arms rearing (Figure 4A) and percentage alternation (Figure 4B) in the HFD/STZ group compared to the control group. Conversely, all PPAR agonist‐treated groups showed a statistically (*p* < 0.05) significant increase in the numbers of open arm entries, duration in open arms, numbers of rearing, and percentage alternation compared to the HFD/STZ group. Moreover, the HFD/STZ/FEN/PIO group showed greater statistically (*p* < 0.05) significant improvement compared to the HFD/STZ/FEN and HFD/STZ/PIO groups.

### 3.4 PPARα/γ agonists improved the oxidant/antioxidant status balance in insulin‐resistant rats

**Figure 5:**
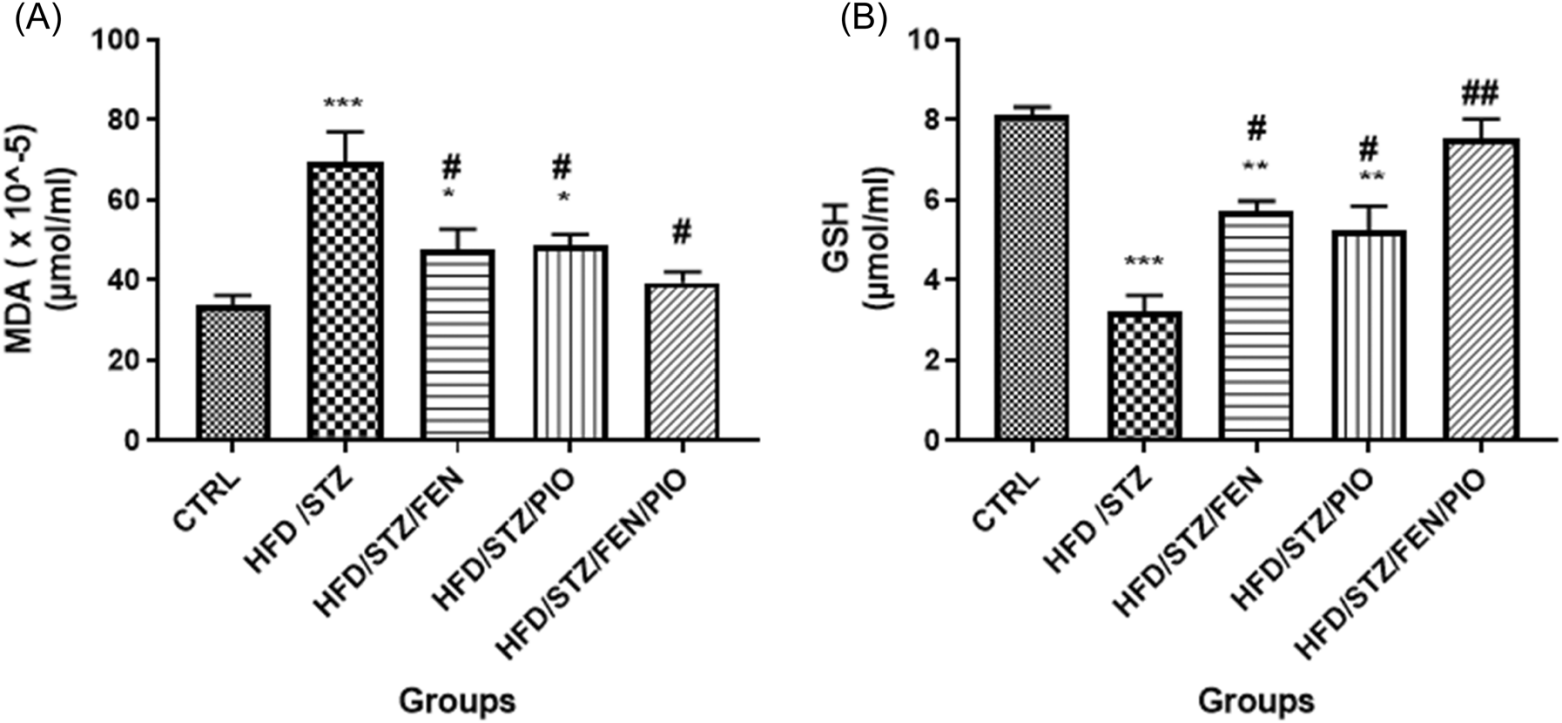
MDA level (A) and GSH level (B). There is a statistically significant increase in MDA levels and decrease in GSH levels in all treated groups, except for the HFD/STZ/FEN/PIO group compared to the control group. All adjuvant‐treated groups showed a statistically significant decrease in MDA levels and increase in GHS levels compared to the HFD/STZ group (*p < 0.05, **p < 0.01, ***p < 0.001 compared to the control group, ^#^ *p* < 0.05, ^##^ *p* < 0.01, compared to the HFD/STZ group. FEN,  fenofibrate; GSH, glutathione; HFD, high‐fat diet; MDA, malondialdehyde; PIO, pioglitazone; STZ, streptozotocin.

There was a significant increase (*p* < 0.001) in MDA levels (Figure 5A) and significantly reduced (*p* < 0.001) GSH levels (Figure 5B) in the HFD/STZ group compared to the control group. On the contrary, all PPAR‐treated groups showed a statistically (*p* < 0.05) significant decrease in MDA levels and an increase (*p* < 0.01) in GSH levels compared to the HFD/STZ group. Furthermore, the HFD/STZ/FEN and HFD/STZ/PIO groups showed significantly higher (*p* < 0.05) MDA levels and lower (*p* < 0.01) GSH levels compared to the control group. Although practically higher MDA and lower GSH values were obtained from the HFD/STZ/FEN/PIO group compared to the control group, there was no statistically significant difference.

### 3.5 PPARα/γ agonists improve hippocampal cellular morphology in insulin‐resistant rats

**Figure 6:**
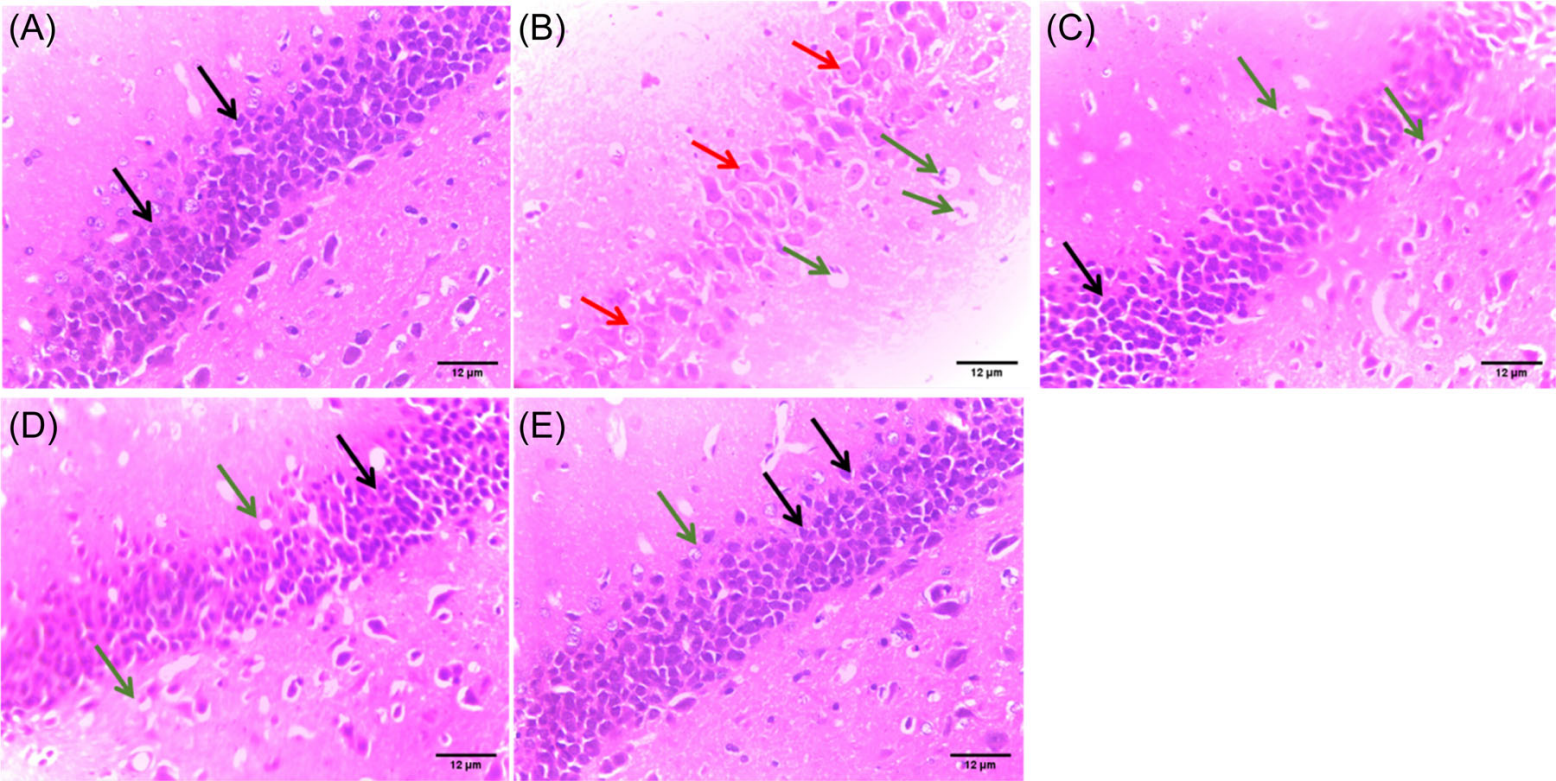
H&E stain histological assessment (×400). (A) Control, (B) HFD/STZ, (C) HFD/STZ/FEN, (D) HFD/STZ/PIO, and (E) HFD/STZ/FEN/PIO groups. (Black arrow: pyramidal neurons, red arrow: pyknotic cells, green arrow: vacuolization). Scale bar = 12 μm. FEN,  fenofibrate; H&E, hematoxylin and eosin; HFD, high‐fat diet; PIO, pioglitazone; STZ, streptozotocin. [Color figure can be viewed at wileyonlinelibrary.com]

Animals fed on normal animal chow (Figure 6A) appeared normal, with the presence of darkly stained pyramidal neurons. The HFD/STZ group (Figure 6B) showed severe cellular alterations, including pyknosis and vacuolization of the pyramidal and vesicular cells. However, the HFD/STZ/FEN and HFD/STZ/PIO groups (Figure 6C,D) showed moderate pathology, with a reduction in nuclei pyknosis and neuronal vacuolization, while the HFD/STZ/FEN/PIO group (Figure 6E) showed only mild pyknosis.

**Figure 7:**
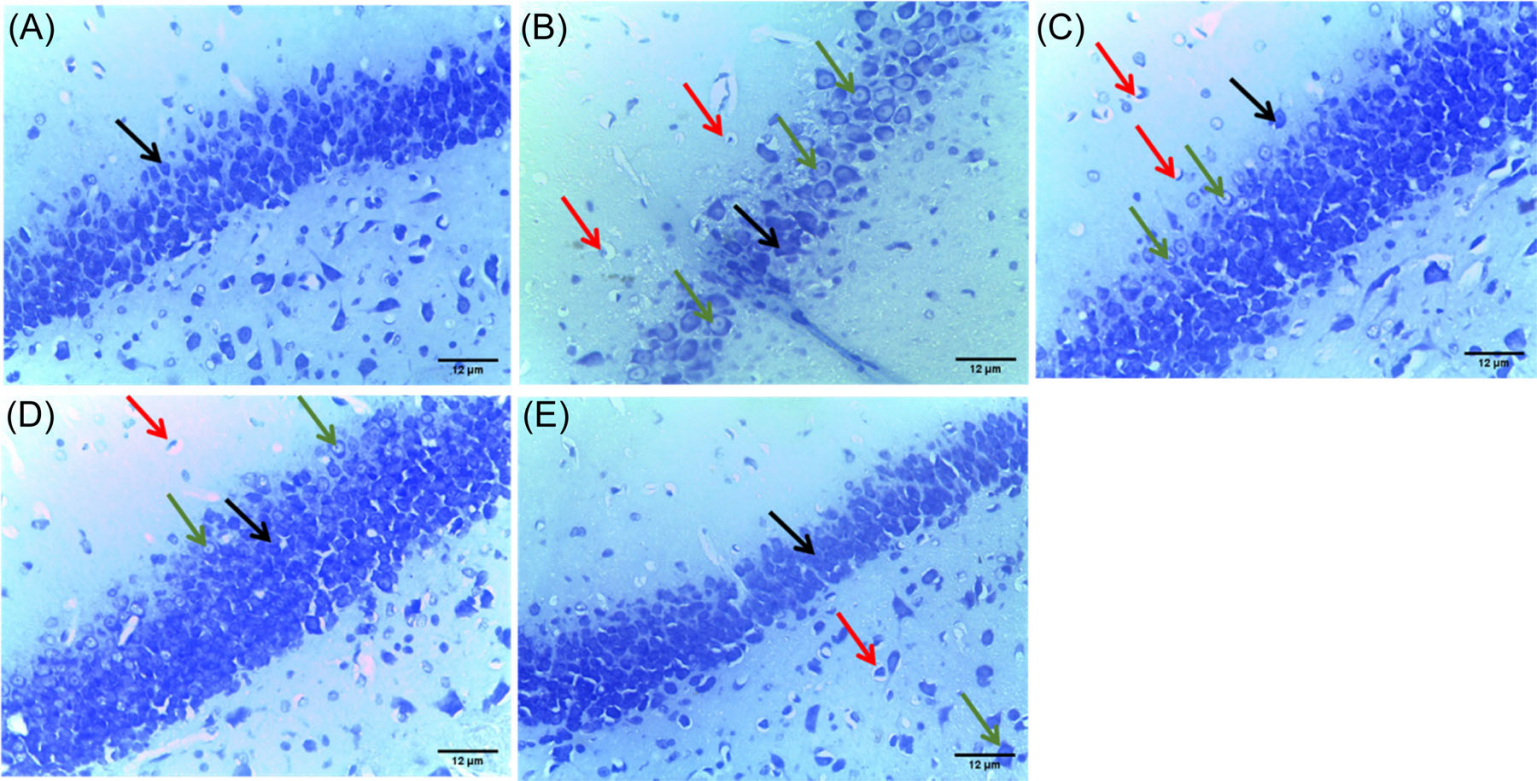
Nissl stain histological assessment (×400). (A) Control, (B) HFD/STZ, (C) HFD/STZ/FEN, (D) HFD/STZ/PIO, and (E) HFD/STZ/FEN/PIO groups. (Black arrow: pyramidal cells, green arrow: chromatolysis). Scale bar = 12 μm. FEN,  fenofibrate; HFD, high‐fat diet; PIO, pioglitazone; STZ, streptozotocin. [Color figure can be viewed at wileyonlinelibrary.com]

Nissl staining showed that the pyramidal cells appeared normal, with the presence of an abundance of nissl substance within the cellular material in the control group (Figure 7A). On the contrary, the HFD/STZ group (Figure 7B) showed a series of nuclei chromatolysis due to the reduction of nissl substance in the nuclei material of the neurons of the hippocampus. The HFD/STZ/FEN, HFD/STZ/PIO, and HFD/STZ/FEN/PIO groups (Figure 7C–E) showed preservation of the nissl material present within the nuclei material of the pyramidal cells.

**Figure 8:**
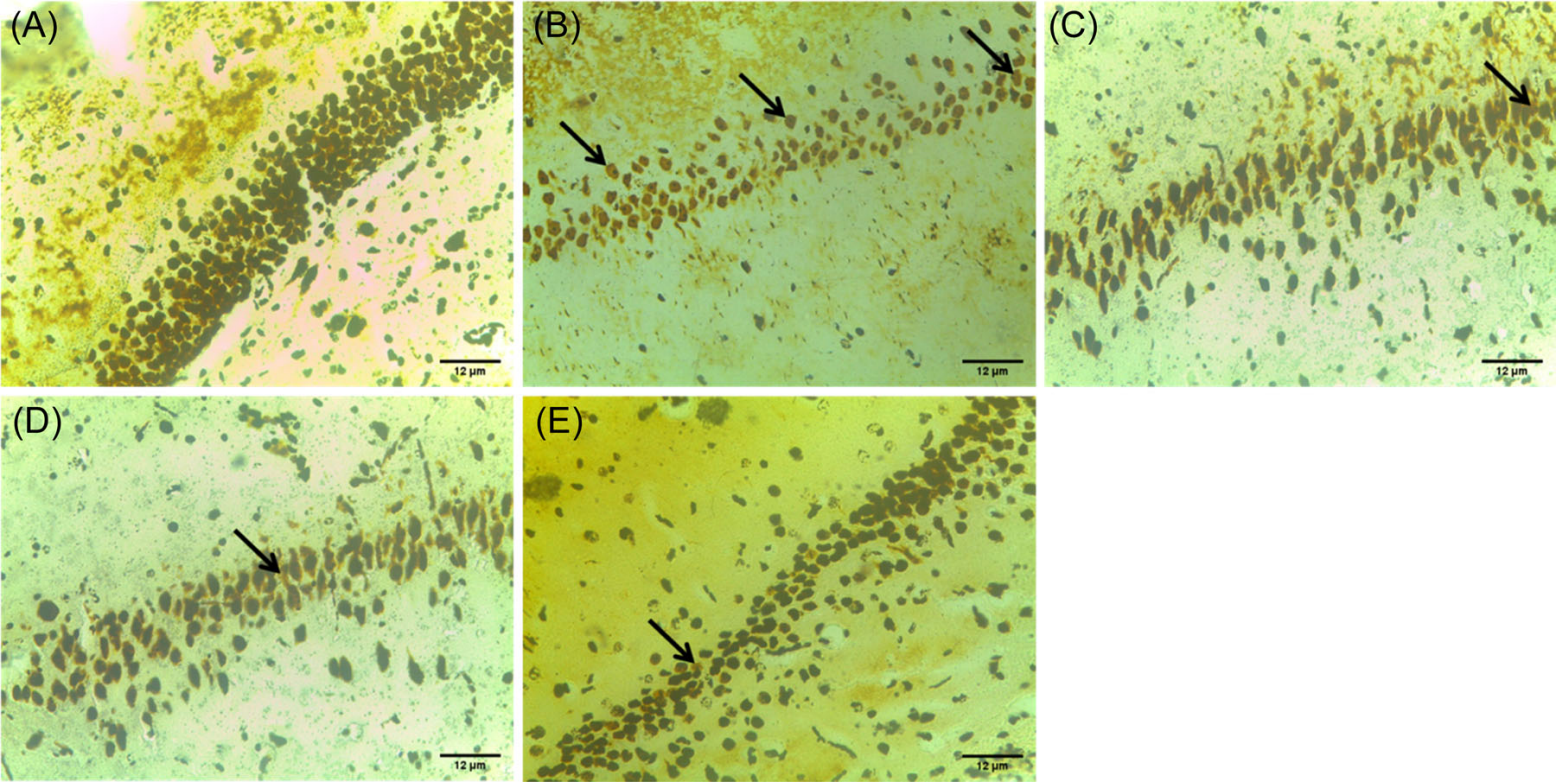
Bielchowsky stain histological assessment (×400). (A) Control, (B) HFD/STZ, (C) HFD/STZ/FEN, (D) HFD/STZ/PIO, (E) HFD/STZ/FEN/PIO groups. (Black arrow: neurofibrils). Scale bar = 12 μm. FEN,  fenofibrate; HFD, high‐fat diet; PIO, pioglitazone; STZ, streptozotocin. [Color figure can be viewed at wileyonlinelibrary.com]

Furthermore, Bielchowsky silver staining showed normal pyramidal neurons, with no to mild traces of neurofibrils in the control group (Figure 8A). The HFD/STZ group (Figure 8B) showed numerous neurofibrils. However, the HFD/STZ/EN and HFD/STZ/PIO groups (Figure 8C,D) showed moderately reduced neurofibrils, while the HFD/STZ/FEN/PIO group (Figure 8E) showed even fewer neurofibrils.

### 3.6 PPARα/γ agonists ameliorate hippocampal neurodegeneration in insulin‐resistant rats

**Figure 9:**
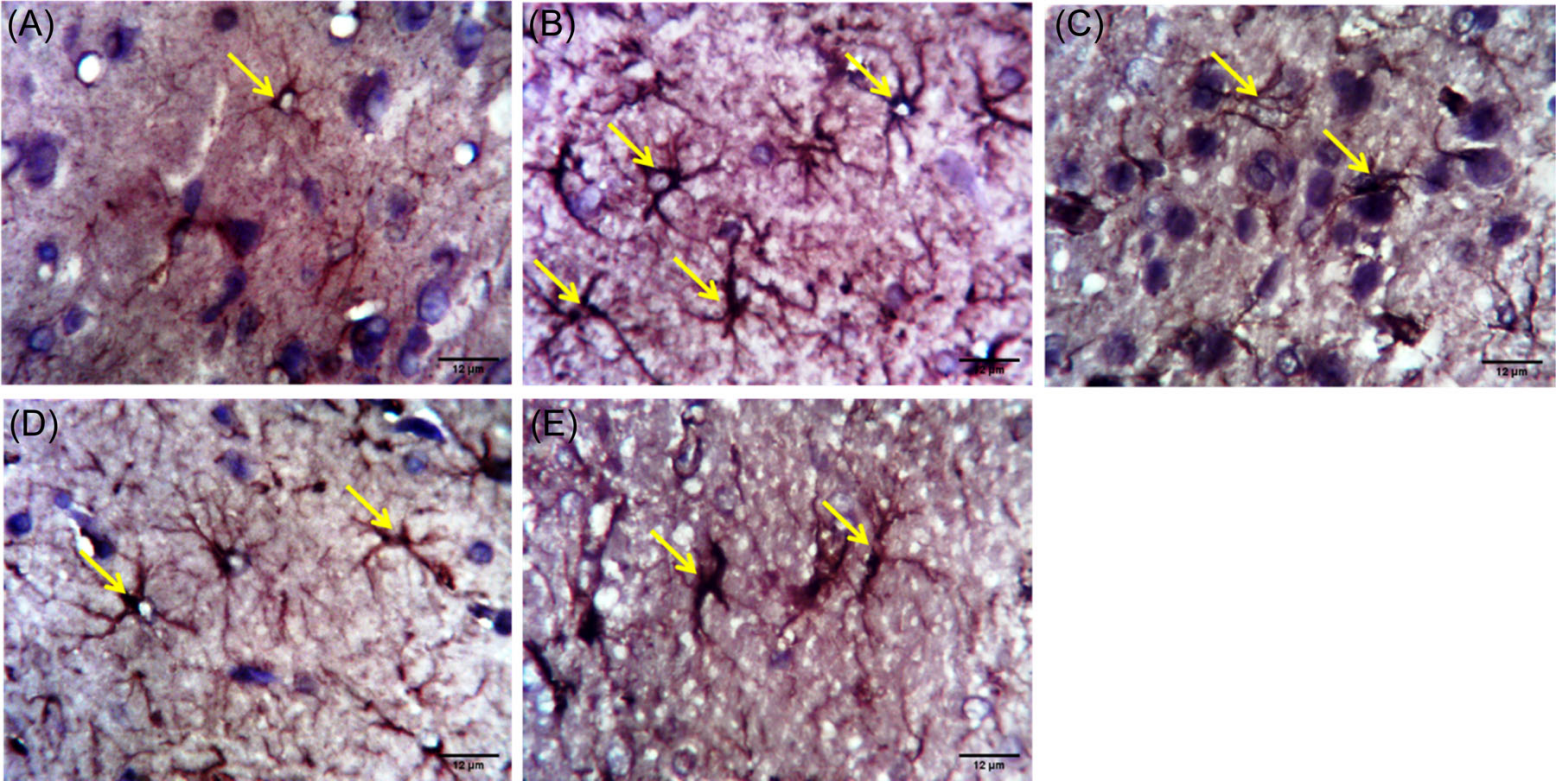
Glial fibrillary acid protein (GFAP) immunohistochemical staining assessment (×400). (A) Control, (B) HFD/STZ, (C) HFD/STZ/FEN, (D) HFD/STZ/PIO, and (E) HFD/STZ/FEN/PIO groups. (Yellow arrow: astrocytes). Scale bar = 12 μm. FEN,  fenofibrate; HFD, high‐fat diet; PIO, pioglitazone; STZ, streptozotocin. [Color figure can be viewed at wileyonlinelibrary.com]

Traces of activated astrocytes in the hippocampal region of the cortex of the control group was observed (Figure 9A). Conversely, dense activation of astrocytes was observed within the pyramidal cell areas of the hippocampus of HFD/STZ groups (Figure 9B). As the control, the HFD/STZ/FEN/PIO group (Figure 9E) showed reduced activation and presence of astrocytes, while the HFD/STZ/FEN and HFD/STZ/PIO groups (Figure 9C,D) showed moderate activation of the astrocytes present at the hippocampal region.

## 4 DISCUSSION

Nowadays, the prevalence of dementia associated with IR‐induced DM is increasing. Evidence of insulin insensitivity triggering neuronal death has been identified as a major mechanism underlying dementia associated with IR.^11^

In the present study, the onset and progressing severity of type 2 DM induced with the use of HFD and STZ induced severe metabolic disorders including elevated FBG levels and insensitivity of the hippocampal cells to blood insulin. Bathina et al. explained that HFD/STZ disrupts the secondary messengers of insulin signaling (pGsk‐3β and Foxo1) and PI3K/Akt/mTOR signaling pathways, thereby reducing the production of ATP required for positive neuroplasticity. Furthermore, the decline in behavioral and cognitive performance in Y‐maze assessment, particularly a decrease in exploratory time in the arms of the Y‐maze and percentage alternation in the arms suggest that HFD/STZ‐induced IR causes a decrease in ATP production needed for memory consolidation and reinforcement of newly formed synaptic connection during the learning phase of the assessment.^46^, ^69^ Therapeutically, PPAR has been described to contribute to the modulation of the secondary messengers of insulin signaling and PI3K/Akt/mTOR.^70^, ^71^ Hence, our findings showed that therapeutic use of FEN (PPARα) and PIO (PPARγ) as monotherapy and combined therapy may significantly improve insulin sensitivity, behavioral response, and cognitive function of the hippocampal neurons via the activation and upregulation of plasma membrane glycolipids and PI3K/Akt/mTOR pathways. Similarly, reduction of free fatty acid and activation of PPARs have been linked to reduced IR needed for glucose control in type 2 DM patients.^72^ Moreover, impaired glucose tolerance and alteration in glucose metabolism have been linked to increased inflammation.^73^, ^74^, ^75^ Therefore, the efficacy of fenofibrate to simultaneously reduce free fatty acids and activate PPARs and insulin sensitization by pioglitazone may be linked to the decreased fasting glucose levels reported in this research. In addition, downstream regulation of STZ‐induced inflammatory cytokines due to the anti‐inflammatory effects of pioglitazone‐ and fenofibrate‐activated PPARs may have improved cellular insulin sensitivity, hence resulting in the decreased fasting glucose level reported in this research. It is noteworthy that the activation of PPARα has been considered to contribute to the upregulation of the mitotic signaling pathway (Wnt‐signaling cascade) of both glial cells and neural progenitor cells in areas of the CNS associated with neurogenesis including the hippocampus.^29^ Hence, another explanation could be that the activation of this mechanism by fenofibrate, coupled with insulin sensitization of the newly populated neurons by Pioglitazone, might have contributed to the improved glucose control, thereby resulting in decreased fasting glucose levels when the therapy was combined.

Evidence of increased reactive oxygen species (ROS) and low antioxidant activities induced by the concomitant use of STZ and HFD suggests that disruption in insulin sensitivity and elevation of FBG induce an imbalance in ROS, hence resulting in deterioration of the activities of antioxidant enzymes and their secretions needed to mop up excess ROS.^46^ Furthermore, a reduction in the sensitivity of the hippocampal neurons to insulin has been linked to the dysregulation of metabolic‐related pathways including mTOR, lactate, and ketone bodies, thereby increasing the production of ROS (increased MDA level) at the cost of the antioxidant enzymes (catalase and SOD) and antioxidant secretions (GSH). Interestingly, the use of PPARα, PPARγ, and PPARα/γ demonstrated the potential to significantly improve antioxidant activities by directly and/or indirectly improving the function of antioxidant enzymes and their secretions. Studies have suggested that the PPAR agonist exerts its antioxidant property by upregulating gene expressions that control ROS detoxification pathways and by reducing cellular superoxide production via optimal alteration of oxidant/antioxidant enzyme activities.^76^, ^77^

Evidence from studies has linked ROS accumulation in conjunction with deterioration in antioxidant functions to the pathophysiology and neurodegeneration associated with IR. The demonstration of severe pathological symptoms associated with neurodegeneration within the cellular morphology of the hippocampus infers that HFD/STZ  may trigger neuronal death as well as impeding synaptic communications essential for cognition. Hence, alterations in the functionality and survival of hippocampal neurons escalated by IR may be the underlying mechanisms contributing to the symptoms of dementia among DM patients. Furthermore, HFD has been reported to upregulate glial glutamate transporters, increasing the expression of GFAP, particularly in cerebral injuries and neurodegeneration disorders.^78^, ^79^ Therefore, evidence of overexpression of astrocytic proteins (GFAP) in HFD/STZ mimicking the symptoms of type 2 DM suggests that IR triggers inflammatory pathways (like the mTOR pathway) within the neurons of the hippocampus, severing the synaptic communication essential for neuroplasticity. Therapeutically, the use of PPARs, particularly the combination of PPARα/γ agonists, enhanced the survival and optimal functioning of the neuron of the hippocampus by mitigating astrogliosis response and inhibiting the inflammatory processes, leading to histological evidence of neuronal death such as pyknosis and vacuolization. Interestingly, there have been controversial reports on the role of PPARs in inflammatory signaling pathways.^80^, ^81^, ^82^, ^83^ However, studies have established that the inflammatory response of PPARs significantly depends on the PPAR activation agents and cell type activated.^84^, ^85^ The use of a synthetic PPARα/γ agonist modifies the conformational organization of activated PPARs located on macrophages including astrocytes to induce anti‐inflammatory activities. Activation of the retinoid X receptor on macrophages by the PPARγ agonist decreases the production of pro‐inflammatory cytokines interleukin‐6 and interleukin‐8 and chemokine ligands (CXCL1, CXCL2, and CXCL3), thereby increasing the efficacy and efficiency of circulating anti‐inflammatory cytokines to induce anti‐inflammatory activities. Moreover, PPARα agonists have been suggested to have a direct modulatory effect on the mRNA expression of anti‐inflammatory cytokines. Therefore, the synergy of these PPARα/γ agonist‐activated PPAR activities may have contributed to the survival and morphology of hippocampal astrocytes and the preservation of cellular conformation of the hippocampus for improved cognition and behavioral performance.

## 5 CONCLUSION

PPARα/γ agonists have been shown to play a vital ameliorative role in hippocampal neurodegeneration. This has been demonstrated in their regulation of inflammatory processes, reduction of oxidative stress, and control of protein aggregation. This study also reveals that combined PPARα/γ agonists have stronger neuroprotective capacities than either PPARα or PPARγ agonists alone.

## AUTHOR CONTRIBUTIONS

Oluwole B. Akinola contributed to the original idea, supervised, and contributed to the scientific content of the research. Olufunke O. Dosumu contributed to the scientific content of the research and proofreading, and contributed to the drafting of the submitted manuscript. Olumayowa K. Idowu and Olushola O. Oluyomi carried out the experimental protocols and drafting of the initial paper. Oluwatomisin O. Faniyan contributed to the data and image analyses of the paper.

## CONFLICT OF INTEREST

The authors declare no conflict of interest.

## ETHICS STATEMENT

Ethical clearance to perform this study with the approval number UERC/ASN/2017/970 was obtained from the Ethical Review Committee of the University of Ilorin, Nigeria, and experimental procedures were conducted in accordance with the guidelines of the National Institute of Health Guide for the Care and Use of Laboratory Animals.

## Data Availability

The authors declare that the data supporting the findings of this study are available from the corresponding author upon reasonable request.
